# MicroRNA-379-5p regulates free cholesterol accumulation and relieves diet induced-liver damage in *db*/*db* mice via STAT1/HMGCS1 axis

**DOI:** 10.1186/s43556-022-00089-w

**Published:** 2022-08-10

**Authors:** Yunxia Dong, Chuwei Yu, Ningning Ma, Xiaoding Xu, Qian Wu, Henglei Lu, Likun Gong, Jing Chen, Jin Ren

**Affiliations:** 1 Center for Drug Safety Evaluation and Research, State Key Laboratory of Drug Research, Shanghai Institute of Materia Medica, Chinese Academy of Sciences, 501 Haike Road, Shanghai, 201203 China; 2 University of Chinese Academy of Sciences, No.19A Yuquan Road, Beijing, 100049 China; 3 School of Life Science and Technology, ShanghaiTech University, 100 Haike Road, Shanghai, 201210 China; 4 School of Chinese Materia Medica, Nanjing University of Chinese Medicine, Nanjing, 210023 China

**Keywords:** Non-alcoholic fatty liver disease, MiR-379-5p, STAT1, HMGCS1, Cholesterol metabolism

## Abstract

Lipotoxicity induced by the overload of lipid in the liver, especially excess free cholesterol (FC), has been recognized as one of driving factors in the transition from non-alcoholic fatty liver (NAFL) to non-alcoholic steatohepatitis (NASH). MicroRNA (miR)-379-5p has been reported to play regulatory roles in hepatic triglyceride homeostasis, but the relationship of miR-379-5p and hepatic cholesterol homeostasis has never been touched. In the current study, we found that hepatic miR-379-5p levels were decreased obviously in NAFLD patients and model mice compared with their controls. Moreover, miR-379-5p was discovered to be able to inhibit intracellular FC accumulation and alleviate mitochondrial damage induced by palmitic acid (PA) in vitro. Furthermore, overexpression of miR-379-5p in HFHC-fed *db/db* mice could reduce the level of hepatic total cholesterol (TC) and FC, and ameliorate hepatic injury reflected by the lower serum alanine aminotransferase (ALT) and aspartate transaminase (AST). Subsequently, by combining spectrometry (MS) and luciferase assay, we identified miR-379-5p suppressed STAT1 through transcriptional and translational regulation. Finally, we confirmed that STAT1 was a transcriptional factor of HMGCS1. In conclusion, miR-379-5p inhibits STAT1 expression and regulates cholesterol metabolism through the STAT1/HMGCS1 axis, suggesting miR-379-5p might be applied to improve lipotoxicity in the future.

**Supplementary Information:**

The online version contains supplementary material available at 10.1186/s43556-022-00089-w.

## Introduction

Non-alcoholic fatty liver disease (NAFLD) is a common wide-spectrum chronic liver disease, ranging from steatosis to non-alcoholic steatohepatitis (NASH), fibrosis or cirrhosis and hepatocellular carcinoma [1]. Its clinical features are manifested by excessive lipids accumulation in the liver without alcohol, drugs, viruses or other known liver damage factors [2]. With the rising prevalence of obesity and weight-related metabolic comorbidities worldwide, NAFLD represents one of the most important causes of liver disease, affecting more than a quarter of the global population, including adults and children [3]. To date, besides lifestyle modification and weight-loss, there has been no effective treatments for NAFLD due to the complex pathogenesis of NAFLD [4].

It is clearly established that lipotoxicity is a critical risk factor to drive hepatic inflammation and subsequent progressive fibrosis [5]. At present, cholesterol, free fatty acids (FFAs) and their derivatives, like diacylglycerol, ceramides, etc. have been accepted as lipotoxic molecules to promote inflammation or fibrosis via desensitizing insulin pathway, inducing hepatocyte apoptosis or impairing membrane function [6]. Liver plays a central role in regulating cholesterol homeostasis. It is well documented that free cholesterol (FC) is accumulated in NASH by reason of extensive dysregulation of hepatic cholesterol metabolism, which leads to the dysfunction of hepatocytes, Kupffer cells (KCs), and hepatic stellate cells (HSCs) [7]. Moreover, the benefits of some cholesterol-modulating drugs, like statins and ezetimibe [8, 9], on NAFLD further suggested inhibiting the overload of hepatic FC might be as an effective therapeutic strategy to alleviate the progression of NAFLD.

MicroRNA (miR)-379 is reported as a tumor-suppressor for its inhibitory effect on cell proliferation and migration in the cancers of the brain, breast, lung, and liver [10–13]. However, the role of miR-379 in metabolic pathways has been discovered by recent studies. Serum miR-379 (miR-379-5p) expression was up-regulated obviously in the patients with early-stage NAFLD, which suggests it might be a biomarker for discriminating NAFLD patients from controls [14]. Another study showed that hepatic miR-379-5p deficiency reduces the level of serum very-low-density lipoprotein-associated triglyceride (VLDL-TG) through promoting hepatic lipid re-uptake and TG accumulation [15]. Recently, a report indicated that lack of miR-379/miR-544 cluster might have important consequences for obesity and fat accumulation [16], in which lack of miR-379/miR-544 cluster in vivo can decrease serum cholesterol. However, the effect of hepatic miR-379 on cholesterol metabolism is still obscure, because this conclusion is deficient in its specificity due to the fact that deleting a huge cluster contains many microRNAs, not only miR-379. Therefore, it is necessary to investigate the effect of hepatic miR-379 on cholesterol metabolism.

In this study, we investigate the role of miR-379-5p in hepatic cholesterol metabolism by in vitro model of huh7 cells induced by palmitic acid (PA) and in vivo model of *db/db* mice induced by high-fat-high-cholesterol (HFHC) diet, and further explored the possible involved pathway.

## Results

### Hepatic miR-379 is downregulated in NAFLD patient and multiple NAFLD models

**Fig. 1:**
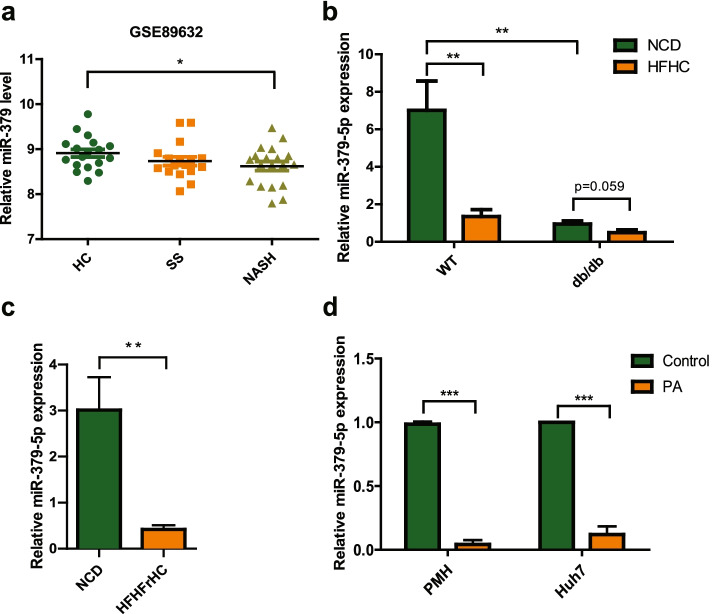
Expression of miR-379-5p is decreased in vitro/in vivo/in clinic. **a** Hepatic miR-379 levels between healthy control (HC, *n* = 18), simple steatosis (SS, *n* = 17) and NASH patients (*n* = 19). Data were derived from GEO database (GSE89632). In HC group, 6 healthy living liver donors with fibrosis symptoms were excluded. **b** *db/db* mice and their lean littermates (wild type (WT)) were fed with a NCD or HFHC diet for 20 weeks respectively, and miR-379-5p in the liver was examined by RT-PCR. *n* = 5 in each group. **c** MiR-379-5p was detected by RT-PCR in the livers of C57BL/6 J mice fed with a NCD or HFHFrHC diet for 20 weeks. *n* = 9–10 in each group. **d** Primary mouse hepatocytes (PMH) and Huh7 cells were stimulated with 0.5 mM PA for 24 h, and the expression of miR-379-5p in the cells was detected by RT-PCR. For a, c and d, two-tailed student’s t-test was used to calculate statistical significance, while for b, One-Way ANOVA test was used (*P < 0.05, **P < 0.01, ***P < 0.001)

As reported, serum miR-379 (miR-379-5p) expression was up-regulated obviously in the patients with early-stage NAFLD. However, its level in the liver has not been mentioned. Since only hepatic miRNAs profile can reflect their function in the liver [17], we examined its expression firstly in the liver from clinical database. As shown in Fig. 1a, the gene array from Gene Expression Omnibus (GEO) databases (GSE89632) (Supplementary Table 1) [18] showed that the hepatic miR-379 levels in NASH patients were decreased obviously versus that in normal subjects (HC). Interestingly, this difference did not exist between simple steatosis (SS) and HC. Then, we detected the level of miR-379-5p in several mouse models. In the *db/db* mice model fed with the HFHC diet or not, hepatic miR-379-5p was down-regulated significantly in the *db/db* mice versus their littermates under the normal feeding condition, and this down-regulation was even more pronounced after the HFHC diet was given in WT group (Fig. 1b). Similarly, this phenomenon is reappeared in the C57BL/6 J mice fed with high-fat/high-fructose/high-cholesterol (HFHFrHC) diet (Fig. 1c). Consistent with our observation in vivo, miR-379-5p expression was decreased significantly after PA treatment in mouse primary hepatocytes (PMH) and Huh7 cells (Fig. 1d). What’s more, we also found serum AST and ALT had the negative correlation with miR-379-5p in NASH patients and mouse models, which indicated miR-379-5p might improve liver damage (Supplementary Fig. 1). These results indicated that hepatic miR-379-5p is correlated with the development of NAFLD.

### Overexpression of miR-379-5p relieves FC accumulation and reduces lipotoxicity in vitro

**Fig. 2:**
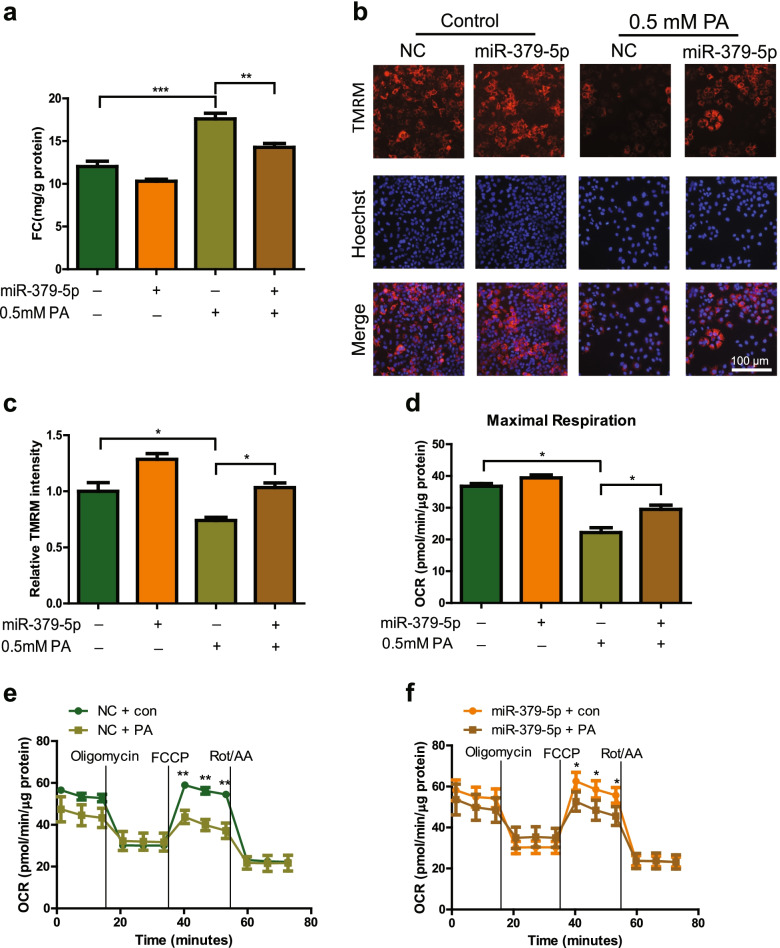
Overexpression of miR-379-5p inhibits PA-stimulated FC accumulation and mitochondrial dysfunction in vitro*.* **a** FC contents in Huh7 cells treated with 0.5 mM PA for 24 h. **b** Representative images of TMRM (red) and Hoechst (blue) in Huh7 cells, Scale bar: 100 μm. **c** Quantitative analysis of TMRM fluorescence intensity in Huh7 cells treated with 0.5 mM PA for 24 h. **d** Seahorse XFe96 analysis of cell maximal respiration in Huh7 cells followed by treatment with 0.5 mM PA or BSA for 24 h. **e** Seahorse XFe96 analysis of cell OCR in Huh7 cells followed by transfected with NC (30 nM) for 48 h and treatment with 0.5 mM PA or BSA for another 24 h. **f** Seahorse XFe96 analysis of cell OCR in Huh7 cells followed by transfected with miR-379-5p mimic (30 nM) for 48 h and treatment with 0.5 mM PA or BSA for another 24 h. One-Way ANOVA test was used to calculate statistical significance. *P < 0.05, **P < 0.01, ***P < 0.001. *n* = 3 independent experiments

Given the FC accumulation in the liver is a significant risk factor for NASH and hepatic miR-379-5p is lower obviously in mouse models, we examined whether miR-379-5p plays regulatory role in cholesterol metabolism. FC content could increase in PA-stimulated Huh7 cells, while overexpression of miR-379-5p can reverse it significantly (Fig. 2a). Due to the FC accumulation can damage mitochondrial function [19, 20], we further investigated whether miR-379-5p can improve the mitochondrial membrane potential, a key indicator of mitochondrial function, after alleviating FC accumulation. The fluorescence intensity of TMRM showed that PA treatment reduced mitochondrial membrane potential significantly, while overexpression of miR-379-5p enhanced the intensity of TMRM and alleviate mitochondrial damage (Fig. 2b-c). Additionally, FC accumulation also can impair mitochondrial respiration and disrupt the assembly of mitochondrial respiratory complex [21, 22]. Thus, the mitochondrial oxygen consumption rate (OCR) was evaluated by Seahorse XFe96 analyzer. As shown in Fig. 2d-f, mitochondrial OCR was suppressed after the treatment with 0.5 mM PA in NC group, while was ameliorated obviously in miR-379-5p group under the same stimulation. These results suggested that miR-379-5p can inhibit FC accumulation and alleviate lipotoxicity-mediated mitochondrial dysfunction.

### Overexpression of miR-379-5p lessens HFHC-induced cholesterol accumulation and ameliorates liver injury in vivo

**Fig. 3:**
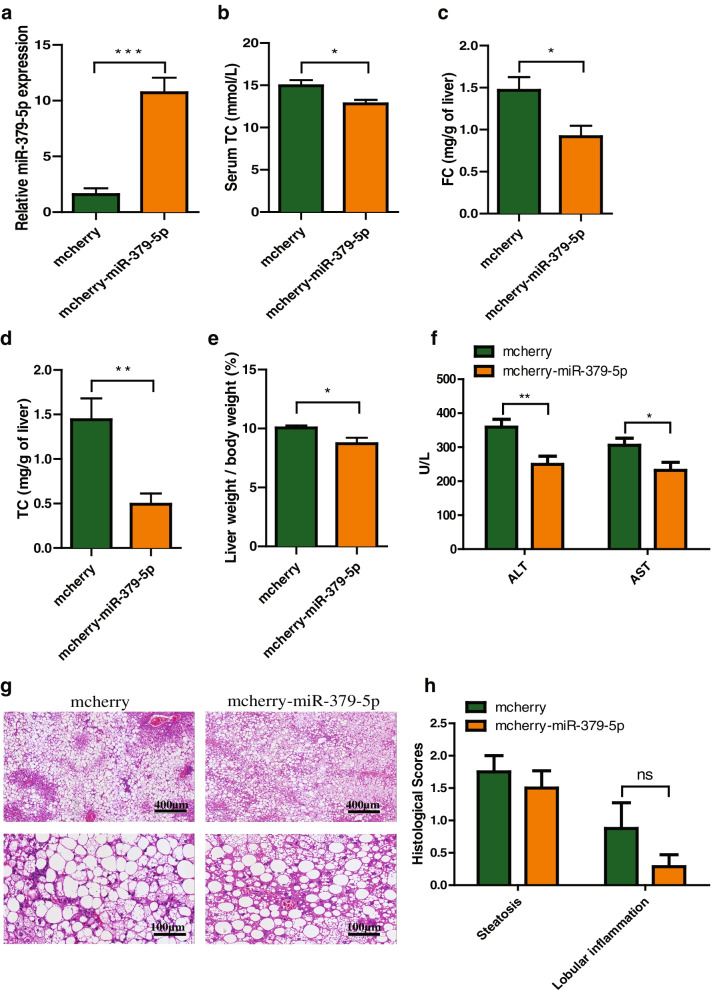
Overexpression of miR-379-5p attenuates the overnutrition-induced cholesterol metabolism disorder and liver damage in *db*/*db* mice. **a** Hepatic miR-379-5p levels after 9 weeks of AAV injection in mcherry and mcherry-miR-379-5p group of *db*/*db* mice with HFHC diet. **b** The serum TC of mcherry and mcherry-miR-379-5p group with HFHC diet. **c** & **d** The hepatic FC and TC of mcherry and mcherry-miR-379-5p group with HFHC diet. **e** The proportion of liver calculated with following formula: Liver weight / body weight × 100% of mcherry and mcherry-miR-379-5p group with HFHC diet. **f** The serum ALT and AST of mcherry and mcherry-miR-379-5p group with HFHC diet. **g** Representative H&E liver sections of mcherry and mcherry-miR-379-5p group with HFHC diet. Scale bar, 400 μm. **h** Histological scores of mcherry and mcherry-miR-379-5p group with HFHC diet. Two-tailed student’s t-test was used to calculate statistical significance, *p < 0.05, **p < 0.01, ***p < 0.001, *n* = 7–8 in each group

Since miR-379-5p can inhibit the lipotoxicity of FC in vitro, we wonder whether this mitigatory effect of miR-379-5p can relieve liver injury in NAFLD mouse model. The mice pre-fed with HFHC for 2 weeks were infected with AAV2/8 carried pre-miR-379. After 9 weeks of AAV injection, hepatic miR-379-5p level was increased significantly in mcherry-miR-379-5p group when compared with its control group (Fig. 3a). Accordingly, the contents of serum TC, hepatic FC and TC were reduced obviously (Fig. 3b-d), and hepatic TG reduced slightly in mcherry-miR-379-5p group (Supplementary Fig. 2a), which indicated that miR-379-5p might exhibit significant improvement in cholesterol accumulation induced by HFHC diet. At the same time, we also found that miR-379-5p overexpression made the weight proportion of liver to body lowered when compared with the mcherry group (Fig. 3e). Moreover, the most widely used biochemical markers for liver injury, serum ALT and AST levels, were markedly decreased in mcherry-miR-379-5p group (Fig. 3f). In addition, H&E staining also showed steatosis between mcherry and mcherry-miR-379-5p group had no difference, which was consistent with the level of hepatic TG. However, lobular inflammation seemed lower in mcherry-miR-379-5p group but without statistical significance (Fig. 3g-h). Notably, we found there were some glycogenated nuclei and megamitochondria in mcherry group, while it was not found in mcherry-miR-379-5p group (Supplementary Fig. 2b). The above results demonstrated that overexpression of miR-379-5p can reduce the hepatic cholesterol accumulation and attenuate liver damage.

### MiR-379-5p inhibits the expression of HMGCS1 without affecting its 3’UTR activity directly

**Fig. 4:**
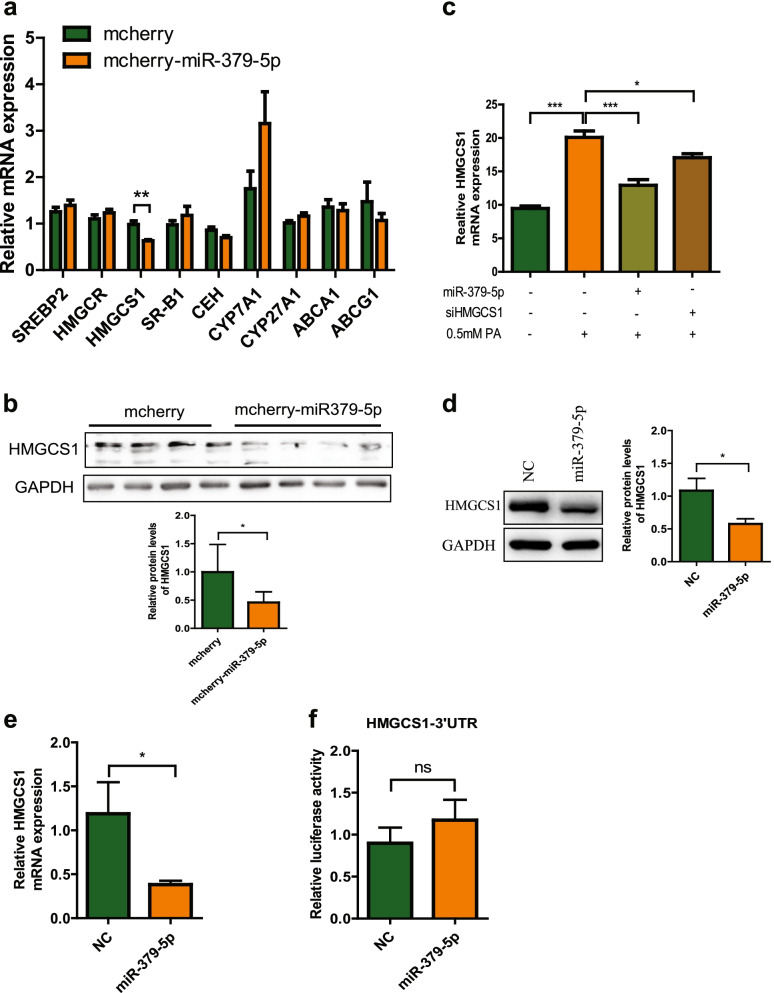
Regulatory effect of miR-379-5p on HMGCS1. **a** The mRNA expression of cholesterol metabolism-related genes (*SREBF2, HMGCR, HMGCS1, SCARB1, CEH, Cyp7a1, Cyp27a1, ABCA1 and ABCG1*) in the livers of HFHC mice, *n* = 7–8 in each group. **b** HMGCS1 protein levels in the livers of HFHC mice, *n* = 7–8 in each group. **c** FC contents in Huh7 cells that were transfected with miR-379-5p mimics (30 nM) or siHMGCS1 (30 nM) for 24 h and then cultured with 0.5 mM PA for another 24 h. **d** & **e** HMGCS1 protein and mRNA levels in Huh7 cells transfected with miR-379-5p mimics (30 nM) or NC (30 nM) for 48 h and then cultured with 0.5 mM PA for another 24 h. **f** The relative activities of HMGCS1 3’UTR presented by relative luciferase activity of HEK293 cells co-transfected with related plasmids and miR-379-5p mimics (30 nM) or NC(30 nM) for 24 h. For a, c, e-f, two-tailed student’s t-test was used to calculate statistical significance, while for b, One-Way ANOVA test was used. *p < 0.05, **p < 0.01, ***p < 0.001, *n* = 3 independent experiments

To explore the possible molecular mechanism underlying the effect of miR-379-5p on free cholesterol accumulation, we detected the change of hepatic mRNAs related to the key genes in cholesterol metabolism between two groups mice by RT-PCR. As shown in Fig. 4a, the mRNA of *HMGCS1*, responsible for cholesterol synthesis [23], was reduced in mcherry-miR-379-5p group significantly compared with the mcherry group, while other genes involved in cholesterol synthesis (*HMGCR*) [24], cholesterol uptake (*SCARB1*) [25], cholesterol esterification (*CEH*) [26], cholesterol conversion (*Cyp7a1*, *Cyp27a1*) [27], and cholesterol efflux (*ABCA1*, *ABCG1*) [28] or key transcription factor, *SREBF2* [29], all had no obvious change. HMGCS1 is a key enzyme in the process of cholesterol synthesis and the protein level of HMGCS1 reduced slightly in mcherry-miR-379-5p group (Fig. 4b), which suggested that miR-379-5p inhibit FC content probably by suppressing HMGCS1. Next, we confirmed this result in Huh7 cells, where PA induced intracellular FC accumulation and miR-379-5p can inhibit this elevation. As same as the effect of miR-379-5p on intracellular FC accumulation, HMGCS1 siRNA also could decrease PA-induced intracellular FC accumulation obviously (Fig. 4c), suggested that HMGCS1 might be a target of miR-379-5p in regulating cholesterol metabolism.

To investigate whether HMGCS1 is regulated by miR-379-5p directly or not, we firstly detected the effects of miR-379-5p on HMGCS1 mRNA and protein levels in Huh7 cells. Compared with NC group, the mRNA and protein levels of HMGCS1 were decreased obviously after transfected with miR-379-5p (Fig. 4d-e). Given the post-transcriptional gene silencing through interacting with the 3’UTR of target mRNA directly is a canonical mechanism of miRNA-mediated gene regulation, we tested the relationship between miR-379-5p and HMGCS1 3’UTR by the dual luciferase report assay, in which the plasmid containing the HMGCS1 3’UTR sequence (psi-HMGCS1–3’UTR) was co-transfected with miR-379-5p mimics or NC into HEK293 cells for 24 h. As shown in Fig. 4f, there was no significant difference in relative luciferase activity of psi-HMGCS1–3’UTR between two groups. Taken together, miR-379-5p can inhibit the expression of HMGCS1 indirectly not via interacting with its 3’UTR.

### MiR-379-5p inhibits STAT1 translation and transcription directly

**Fig. 5:**
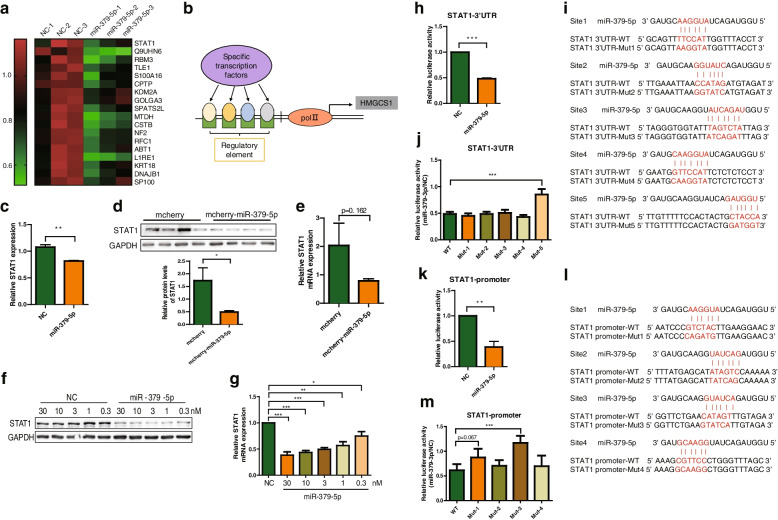
Regulatory effect of miR-379-5p on STAT1. **a** Heatmap for 18 down-regulated proteins in the nucleus of miR-379-5p (30 nM)-treated Huh7 cells, compared to NC (30 nM)-treated Huh7 cells. **b** Schematic diagram of predicted transcription factors of HMGCS1. **c** Relative STAT1 protein level between miR-379-5p- and NC-transfected cells obtained from the data of MS. **d** & **e** STAT1 protein and mRNA levels in the livers of HFHC mice, *n* = 7–8 in each group. **f** & **g** STAT1 mRNA and protein levels in Huh7 cells transfected with different concentrations of miR-379-5p mimics or NC for 72 h. **h** STAT1 3’UTR activity in miR-379-5p (30 nM)- and NC (30 nM)-transfected HEK293 cells. **i** Five putative binding sequence between miR-379-5p and STAT1 3’UTR and their related mutants. **j** The activity of WT or mutants of STAT1 3’UTR in miR-379-5p (30 nM)- and NC (30 nM)-transfected HEK293 cells. **k** STAT1 promoter activity in miR-379-5p (30 nM)- and NC (30 nM)-transfected HEK293 cells. **l** Four putative binding sites between miR-379-5p and STAT1 promoter and their related mutants. **m** The activity of WT or mutants of STAT1 promoter in miR-379-5p (30 nM)- and NC (30 nM)-transfected HEK293 cells. For c, f and i, two-tailed student’s t-test was used to calculate statistical significance, while for d, h and k, One-Way ANOVA test was used. *p < 0.05, **p < 0.01, ***p < 0.001, *n* = 3 independent experiments

To further clarify how miR-379-5p inhibits the expression of HMGCS1 indirectly, we firstly speculated that miR-379-5p might influence the transcription factor in charge of HMGCS1 transcription. After obtaining the information of differentially expressed proteins in the nucleus induced by miR-379-5p via Tandem Mass Tag (TMT)-based quantitative proteomics and cellular component (GOCC) analysis, we found 18 proteins were down-regulated significantly in the nucleus (log2 ratio > 1.2, *p* < 0.05, FDR < 0.01) (Fig. 5a). Subsequently, we used the open-access database (hTFtarget) [30] to predict the possible transcription factors (TFs) that might bind to HMGCS1 promoter region (Fig. 5b). According to the intersection of MS and predictions results, we found that STAT1 meets these two requirements at the same time (Fig. 5c). Coincidentally, STAT1 has been reported to play regulatory role in the process of NAFLD [31], which motivated us to investigate whether miR-379-5p-regulated HMGCS1 is dependent through its effect on inhibiting the expression of STAT1.

Then, we detected the effect of miR-379-5p on the expression of STAT1 in vivo and in vitro to verify this hypothesis. As shown in Fig. 5d-e, STAT1 protein levels were decreased in mcherry-miR-379-5p group, while its mRNA showed a decreasing trend without statistical significance due to the great differences among individuals in mcherry group. In Huh7 cells, STAT1 protein and mRNA levels reduced in a dose-dependent manner after transfected with miR-379-5p (Fig. 5f-g). Next, we constructed the luciferase report plasmid containing the 3’UTR sequence (psi-STAT1–3’UTR) of STAT1 to explore the mechanism of miR-379-5p on STAT1. Luciferase assay shown that miR-379-5p inhibited the relative luciferase activity of psi-STAT1–3’UTR significantly compared with NC (Fig. 5h), indicating that it can suppress the translation of STAT1.

For the reason of figuring out the interaction sites between miR-379-5p and STAT1 3’UTR, we aligned their sequences based on the principle of complementary base pairing and five consecutive possible complementary sequences with a length of six or seven nucleotides on STAT1 3’UTR were found. Then, we performed related luciferase assay with the mutants of these matched sequences. As illustrated in Fig. 5i-j, the inhibitory effect of miR-379-5p on Mut-5 was restored while it still existed on Mut-1, Mut-2, Mut-3, and Mut-4. That is to say, miR-379-5p could bind to the site 5 on the 3’UTR of STAT1 to inhibit its translation.

Due to transcriptional gene regulation has also been discovered as a non-canonical mechanism of miRNA-mediated gene regulation [32], we also investigated the effect of miR-379-5p on STAT1 promoter. The luciferase report plasmid containing the STAT1 promoter sequence (pGL-STAT1-promoter) was used to explore the function of miR-379-5p on STAT1 promoter. As shown in Fig. 5k, miR-379-5p inhibited the relative luciferase activity of pGL-STAT1-promoter significantly compared with NC, indicating that it can suppress the transcription of STAT1. The similar strategy was used to find the interaction site between miR-379-5p and STAT1 promoter. The result shown in Fig. 5l demonstrated that miR-379-5p has four possible binding sites with STAT1 promoter, among which site 3 might be the miR-379-5p-binding site on STAT1 promoter due to that the inhibitory effect of miR-379-5p on this mutant (Mut-3) had abolished (Fig. 5m). To sum up, miR-379-5p could match to STAT1 3’UTR and promoter to inhibit the expression of STAT1.

### Inhibition of STAT1 suppresses the transcription of HMGCS1

**Fig. 6:**
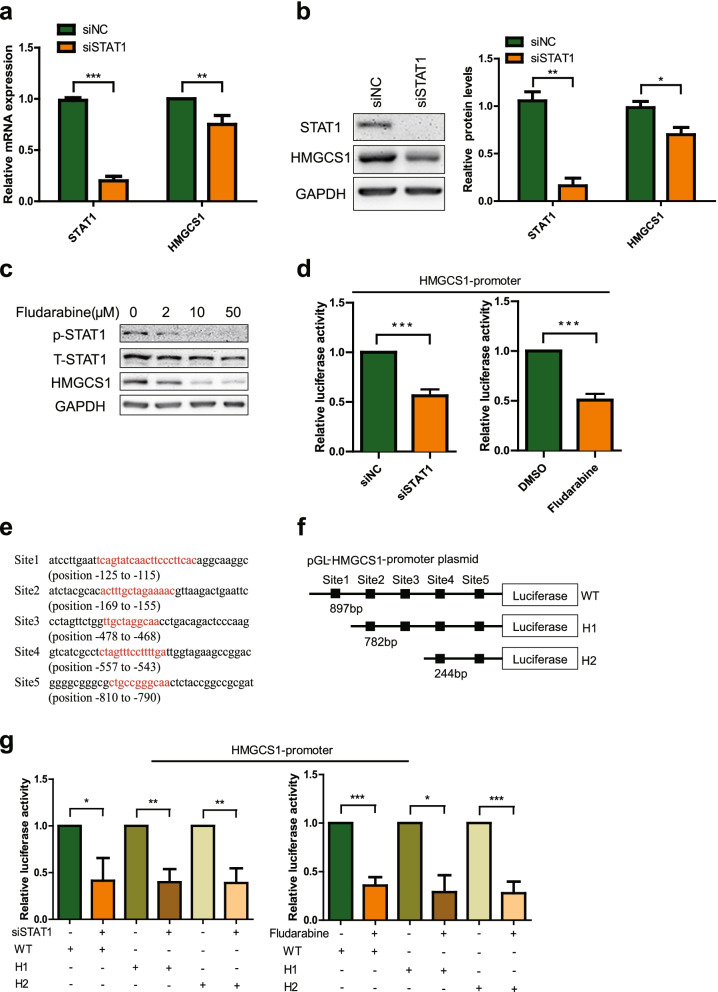
Regulatory effect of STAT1 on HMGCS1. **a** & **b** STAT1 and HMGCS1 mRNA and protein levels in Huh7 cells transfected with siSTAT1 (30 nM) or siNC (30 nM) for 72 h. **c** The protein levels of STAT1 and HMGCS1 in Huh7 cells treated with different concentrations of Fludarabine for 24 h. **d** Relative activity of HMGCS1 promoter in HEK293 cells under the condition of siSTAT1 (30 nM) transfection or Fludarabine (10 μM) treatment. **e** Five binding sequences between STAT1 and HMGCS1 promoter predicted by the JASPAR. **f** Schematic diagram of HMGCS1 promoter (WT) and related truncations (H1 and H2). **g** Relative luciferase activity of HMGCS1 promoter WT, H1 and H2 in HEK293 cells transfected with siSTAT1 (30 nM) or treated with Fludarabine (10 μM) for 24 h. Two-tailed student’s t-test was used to calculate statistical significance. *p < 0.05, **p < 0.01, ***p < 0.001, *n* = 3 independent experiments

Since there has been no report about STAT1 is a transcription factor of HMGCS1 yet, we used siSTAT1 and specific STAT1 inhibitors (Fludarabine) to confirm the effect of STAT1 on HMGCS1 mRNA and protein levels. The mRNA and protein levels of STAT1 and HMGCS1 were reduced significantly after siSTAT1 treatment (Fig. 6a-b). Similarly, the protein level of HMGCS1 was down-regulated significantly after Fludarabine treatment (Fig. 6c).

Next, we examined the regulatory effect of STAT1 on HMGCS1 promoter. As shown in Fig. 6d, siSTAT1 and Fludarabine inhibited the relative luciferase activity of pGL-HMGCS1-promoter significantly, indicating that decreased expression or activity of STAT1 can inhibit the transcription of HMGCS1.

Finally, we predicted and analyzed the possible binding sites of STAT1 on HMGCS1 promoter region through the JASPAR database [33]. According to the prediction results, the related truncated pGL-HMGCS1-promoter were constructed (pGL-HMGCS1-promoter-H1 and pGL-HMGCS1-promoter-H2) (Fig. 5e-f). Similarly, we examined the influence of siSTAT1 and Fludarabine on the relative luciferase activity of pGL-HMGCS1-promoter-H1 and pGL-HMGCS1-promoter-H2. The results showed that the relative luciferase activity of pGL-HMGCS1-promoter-H1 and pGL-HMGCS1-promoter-H2 were still decreased significantly when STAT1 was knockdown or its activity was inhibited (Fig. 6g). The above results indicate that there are multiple binding sites between STAT1 and the promoter region of HMGCS1.

## Discussion

The lipotoxicity caused by the increase of FC has become an important pathogenic factor that causes the occurrence and development of NAFL/NASH [34]. In mitochondria, overload of FC alters mitochondrial membrane fluidity and weakens the function of 2-oxoglutarate carrier in charge of transporting of glutathione from the cytosol into the mitochondria and controlling reactive oxygen species (ROS) generation. The reduction of glutathione in mitochondria promotes ROS production, lipid peroxidation, and hepatocyte necrosis and apoptosis [35]. In the current work, we firstly discovered that hepatic miR-379-5p is lower in NAFLD from clinical database and experimental data of murine/cell model, and the positive effect of miR-379-5p on excessive FC accumulation and mitochondrial function in PA-induced hepatocyte. Further animal experiment also confirmed that miR-379-5p play a hepatoprotective effect via alleviating hepatic FC accumulation effectively.

STAT1 is an important transcription factor that connects cell membrane receptors and effectors for signal transduction. STAT1 in the cytoplasm can be phosphorylated and aggregated to form homodimers or heterodimers under the stimulation of extracellular signals, and then enters the nucleus to promote target gene transcription [36]. It has been reported that STAT1 plays an important role in inducing liver inflammation and damage [31, 37]. Moreover, STAT1 signaling is elevated in the livers of NAFLD model mice and obese patients with NAFLD [38]. Therefore, inhibiting the signal pathway of STAT1 can improve inflammation and liver damage. In our work, we provide evidence to suggest that miR-379-5p negatively regulates STAT1 expression through transcriptional and post-transcriptional levels, showing the function of improving liver injury. HMGCS1 is one of the key enzymes in FC biosynthesis, and inhibiting its expression can reduce the FC synthesis pathway [39]. It has been reported that HMGCS1 is mainly regulated by SREBP2. In addition to SREBP2, our research found another transcription factor, STAT1, to regulate the transcription of HMGCS1. Inhibition of STAT1 can down-regulate the expression of HMGCS1, reduce FC biosynthesis and alleviate FC accumulation.

As we all know, miRNA usually exerts its function in repressing gene expression at post-transcription level [40]. However, other regulatory manners, such as transcriptional regulation via interacting with the promoter or cofactors of transcription factors in the nucleus is also been found [41, 42]. Additionally, the different region of miRNA all can be involved in regulating target expression. The multiple action modes and action regions determine the diversity of miRNA targets, which indicated the possible advantages of miRNA in alleviating metabolic disorders caused by polygenic changes.

Through KEGG analysis of the TMT-based quantitative proteomics, miR-379-5p also can up-regulate 28 proteins involved in metabolic pathways and down-regulate 3 proteins involved in biosynthesis of unsaturated fatty acids (Supplementary Table 2), which might contribute to its hepatoprotective effect on HFHC mice and needs further investigation. Moreover, NAFLD animal models do not completely mirror the human disease, thus more experimental verification is needed.

In summary, miR-379-5p can inhibit the expression of STAT1 through both transcriptional and translational levels, and then down-regulate the STAT1/HMGCS1 axis to improve FC overload, which suggested that miR-379-5p might be a novel regulator in cholesterol metabolism and improve liver damage in NAFLD.

## Materials and methods

### Animal experiments

All animal protocols were carried out according to the Institutional Ethical Guidelines on animal care and approved by the Institutional Animal Care and Use Committee of the Shanghai Institute of Materia Medica, Chinese Academy of Sciences (2019–04-RJ-191 and 2020–04-RJ-213). All mice were housed in a specific pathogen free (SPF) facility (23 ± 1 °C, 12 hours light/12 hours dark cycles, 50% relative humidity) and given free access to food and water.

Two murine models of NAFLD were used to examine the expression of hepatic miR-379-5p during the development of NAFLD. One is eight-week-old male C57BL/6 J mice (Shanghai Jihui Laboratory Animal Care Co., Ltd., China) fed with a high-fat/high-fructose/high-cholesterol (HFHFrHC) diet (2% cholesterol by weight and 40% of calories derived from fat, 20% from fructose, Research Diets, USA) for 20 weeks (*n* = 9–10 in each group), and the other is six-week-old male *db/db* mice and their littermates (GemPharmatech Co., Ltd., China) fed with a normal control diet (NCD) (24.02% of calories derived from protein; 63.03% from carbohydrate; 12.95% from fat. Beijing KeAo XieLi feed co., ltd, China) or HFHC diet (71.5% Purina Rodent Chow; 0.5% Cholesterol; 5% Fructose; 11.5% Coconut Oil; 11.5% Corn Oil by weight. Research Diets, USA) for 20 weeks (each group *n* = 5). Mice fed a NCD served as controls. Then, the mice were sacrificed after overnight fasting and liver tissues were collected for subsequent analysis.

For miR-379-5p functional investigation, six-week-old male *db/db* mice (GemPharmatech Co., Ltd., China) were fed with HFHC diet for 2 weeks and then injected with adeno-associated viruses intravenously (AAV2/8, 1 × 10^12^ viral particles per mouse) via tail vein after grouping according to the mean of body weight (mcherry and mcherry-miR-379-5p, *n* = 10 in each group). Mice were infected with GPAAV-Mcherry for mcherry group or GPAAV-Mcherry-miR-379 containing the sequence of pre-miR-379 for mcherry-miR-379-5p group. Next, the mice continued to be raised by HFHC diet until the end of experiment. When mice were sacrificed, their tissue samples were snap-frozen in liquid nitrogen and stored at − 80 °C.

### Biochemical analysis

Alanine aminotransferase (ALT), aspartate transaminase (AST) and total cholesterol (TC) in the serum starved overnight were assayed with Roche Cobas C501 automatic biochemistry analyzer. Hepatic TC and FC were detected with Amplex™ Red Cholesterol Assay Kit (Molecular Probes, Invitrogen) under the instruction of the kit.

### Cell culture

Huh7 cells, HEK293 cells and 293 T cells were cultured in DMEM (HyClone, USA) containing with 10% FBS (Gibco, USA) and 1% penicillin-streptomycin (Thermo Fisher Scientific, USA). All cells were grown in a humidified atmosphere of 5% CO_2_, 37 °C.

Primary hepatocytes were isolated from anesthetized male mice according to the standard protocol as previously described [43]. The obtained hepatocytes were resuspended in Williams’ E medium (contain 10% FBS, 1% penicillin-streptomycin, 1% L-glutamine, 1% 1 M HEPES, 1% Insulin-Transferrin-Selenium (ITS, Thermo Fisher Scientific)) and seeded in plates coating with rat tail collagen. The primary hepatocytes and Huh7 cells were stimulated by 0.5 mM of PA for 24 h to establish in vitro models of NAFLD.

### Adeno-associated virus construction

The empty adeno-associated virus plasmid (GPAAV-Mcherry) was purchased from Genomeditech (Shanghai, China). The pre-miR-379 sequence and its flanking DNA sequence were amplified from the genome DNA of mouse primary hepatocytes and inserted into the GPAAV-Mcherry plasmid through seamless cloning to obtain GPAAV-Mcherry-miR-379 plasmid which overexpressed miR-379-5p in mouse liver. The integrity of the sequence was evaluated by DNA sequencing. The GPAAV-Mcherry plasmid served as a control.

The 293 T cells were used for the package of AVV by co-transfecting GPAAV-Mcherry-miR-379 (or GPAAV-Mcherry), pHELPER and pAAV2/8 (1:1:1) via the method of calcium phosphate-based cell transfection. The AVV was purified according to the published approach [44] and AAV titers were determined by RT-PCR with vector-specific primers.

### Plasmids construction

The cDNA and genome DNA of Huh7 cells were prepared for the construction of reporter plasmids of related 3’UTR and promoter. The HMGCS1 3′-untranslated region (3’UTR) (NM_001098272.3, 3661 bp) and STAT1 3’UTR (NM_001384880.1, 1554 bp) were amplified and inserted into the psiCHECK2 (Promega, USA) with Xhol and BamH1. The STAT1 promoter sequence (− 1500 to + 5) was amplified and inserted into the pGL-3Basic (Promega, USA) with Kpn1 and Xhol1. The related mutants were generated by MutanBEST Kit (TaKaRa Biotechnology, Japan). The integrity of the sequence was evaluated by DNA sequencing.

### Cell transfection

The plasmids transfection or plasmids and miRNA mimics co-transfection were performed using Lipofectamine 2000 (Invitrogen, USA), and small interfering RNA (siRNA) or miRNA mimics transfection were using Lipofectamine RNAiMAX (Invitrogen, USA). miRNA mimics were purchased from GenePharma Co., Ltd. (China), and STAT1 siRNA was from by RiBoBio (Guangzhou, China). The siRNA sequences were listed as follows: siSTAT1: 5′- CTGGATATATCAAGACTGA − 3′; siHMGCS1: 5′- GGAACGTGGTACTTAGTTA-3′; siNegative Control: 5′-UUCUCCGAACGUGUCACGUTT − 3′.

### Luciferase assay

For 3’UTR luciferase reporter: After cultured in 96-well plates overnight, HEK293 cells were co-transfected with luciferase reporter plasmids (psiCHECK-HMGCS1–3’UTR or psiCHECK-STAT1–3’UTR, 100 ng/well) and miRNA mimics for 24 h. Then cells were lysed for subsequent luciferase assay by the Dual-Luciferase Reporter Assay System (Promega, USA) and firefly luciferase activity was detected under the guideline of the manufacturer’s instructions.

For promoter luciferase reporter: The luciferase reporter plasmids (pGL-3Basic-STAT1-promoter, 100 ng/well) and Renilla Luciferase vector (SV40, 20 ng/well) were transfected together with miRNA mimics into HEK293 cells for 24 h. Then cells were lysed for subsequent luciferase assay and normalized to Renilla luciferase activity.

### RNA extraction and real-time quantitative PCR analysis

Total RNA was isolated from liver tissues or cell samples with RNAiso Plus reagent (Takara, Japan) and reverse transcribed to cDNA with PrimeScript™ RT Master Mix (Takara, Japan). Real-time quantitative polymerase chain reaction (RT-qPCR) analysis was performed using TB Green PCR Kit (Yeasen, China) by 7500 Fast Real-Time PCR System (Applied Biosystems). GAPDH was used to normalize the gene expression of each sample. The following primers were listed as follows: hSTAT1, forward: 5′- CAGCTTGACTCAAAATTCCTGGA − 3′, reverse: 5′- TGAAGATTACGCTTGCTTTTCCT − 3′, hHMGCS1, forward: 5′- CTCTTGGGATGGACGGTATGC − 3′, reverse: 5′- GCTCCAACTCCACCTGTAGG − 3′, hGAPDH, forward: 5′- GGAGCGAGATCCCTCCAAAAT − 3′, reverse: 5′- GGCTGTTGTCATACTTCTCATGG − 3′. mSREBF2, forward: 5′- GCAGCAACGGGACCATTCT − 3′, reverse: 5′- CCCCATGACTAAGTCCTTCAACT − 3′, mHMGCR, forward: 5′- AGCTTGCCCGAATTGTATGTG − 3′, reverse: 5′- TCTGTTGTGAACCATGTGACTTC − 3′, mHMGCS1, forward: 5′- AACTGGTGCAGAAATCTCTAGC − 3′, reverse: 5′- GGTTGAATAGCTCAGAACTAGCC − 3′, mSCARB1, forward: 5′- AAACAGGGAAGATCGAGCCAG − 3′, reverse: 5′- GGTCTGACCAAGCTATCAGGTT − 3′, mCEH, forward: 5′- TTGAATACAGGCTAGTCCCACA − 3′, reverse: 5′- CAACGTAGGTAAACTGTTGTCCC − 3′, mCyp7a1, forward: 5′- GGGATTGCTGTGGTAGTGAGC − 3′, reverse: 5′- GGTATGGAATCAACCCGTTGTC − 3′, mCyp27a1, forward: 5′- GCACAGGAGAGTACGGAGG − 3′, reverse: 5′- CGGGCAAGTGCAGCACATA − 3′, mABCA1, forward: 5′- GCTTGTTGGCCTCAGTTAAGG − 3′, reverse: 5′- GTAGCTCAGGCGTACAGAGAT − 3′, mABCG1, forward: 5′- CTTTCCTACTCTGTACCCGAGG − 3′, reverse: 5′- CGGGGCATTCCATTGATAAGG − 3′, mGAPDH, forward: 5′- AGGTCGGTGTGAACGGATTTG − 3′, reverse: 5′- GGGGTCGTTGATGGCAACA − 3′.

For the quantification of miR-379-5p, total RNA was reverse transcribed using TaqMan™ MicroRNA Reverse Transcription Kit (Thermo Fisher Scientific) and RT-qPCR analysis was conducted using TaqMan Universal Master Mix II (Thermo Fisher Scientific) by 7500 standard RT-PCR System. The U6 (snRNA) was used for normalization. TaqMan probes U6 and miR-379-5p primer were purchased from Invitrogen.

### Western blot analysis

The isolation, quantification and western blot of related proteins were according to the protocol as previously described [45]. GAPDH (2118S, Cell Signaling Technology, USA) was selected as the internal control. Additionally, other antibodies used in this study were included STAT1 (9172S, Cell Signaling Technology, USA), p-STAT1 (AP0054, ABclonal Biotechnology Co., Ltd) and HMGCS1 (A3916, ABclonal Biotechnology Co., Ltd).

### Mitochondrial membrane potential analysis

Tetramethylrhodamine methyl ester (TMRM) fluorescent probe was used to represent mitochondrial membrane potential. The nuclear dye Hoechst 33258 was used to define cell count. The relative mitochondrial membrane potential was defined as total TMRM fluorescence intensity of the whole cell area divided by cell count. After treatment by miRNA mimics and washing by PBS, the cells were incubated with two fluorescence probes (Hoechst 33258, 1.5 μg/mL; TMRM, 100 ng/mL) simultaneously for 30 min. Following washed three times with PBS, the fluorescence images of cells were photographed using the Operetta CLS™ (PerkinElmer, USA) and quantified using Harmony Software, in which the 20× objective was used to collect at least 1000 cells for each fluorescence channel.

### Cell oxygen consumption rate analysis

Huh7 cells were cultured in Seahorse 96-well plates (Agilent Technologies, USA) with 6000 cells in each well and transfected with miRNA mimics for 48 h. The cells were exposed to 0.5 mM PA for an additional 24 h. Then, the medium was replaced with 180 μL unbuffered Seahorse XF DMEM medium (pH 7.4) supplemented with glucose (1 mM), pyruvate (100 mM), glutamine (200 mM) equilibrated at 37 °C in a CO_2_-free incubator for 1 h following manufacturer’s instructions. Respiration was expressed as oxygen consumption rate (OCR, pmol/min). 1.5 μM oligomycin was used to inhibit the F0/F1 ATPase activity, 0.5 μM FCCP was acted as a mitochondrial electron transport chain uncoupler, and 0.5 μM rotenone & antimycin A were used to inhibit activity of the complexes I and III, respectively. These mitochondrial inhibitors were added during the assay and used to determine mitochondrial function parameters. OCR was normalized to the protein content of each well for all measurements by BCA assay [46].

### Statistical analysis

GraphPad Prism software was used to analyze and plot the experimental data. Data were shown as the mean ± SEM in vivo and mean ± SD in vitro. The difference between two groups was analyzed by Two-tailed student’s t-test, and more than two groups by One-Way ANOVA test. *p < 0.05, **p < 0.01, ***p < 0.001 were considered statistically significant*.*

## Supplementary Information

**Additional file 1: Supplementary Fig. 1.** MiR-379 negatively correlated with serum AST and ALT in clinic and mouse models. **Supplementary Fig. 2.** The TG content and H&E staining in *db*/*db* mice. **Supplementary Table 1.** Baseline characteristics of the study participants in GSE89632. **Supplementary Table 2.** MiR-379-5p regulates proteins involved in metabolism through KEGG analysis of the TMT-based quantitative proteomics.

## Data Availability

The data of this study are available from the corresponding author on reasonable request.

## Abbreviations

AAV: Adeno-associated viruses
ALT: Alanine aminotransferase
AST: Aspartate transaminase
ChIP: Chromatin immunoprecipitation
FC: Free cholesterol
FFAs: Free fatty acids
GEO: Gene Expression Omnibus
HFHC: High-fat/high-cholesterol
HFHFrHC: High-fat/high-fructose/high-cholesterol
HSCs: Hepatic stellate cells
KCs: Kupffer cells
MS: Mass spectrometry
NAFLD: Nonalcoholic Fatty Liver Disease
NAFL: Nonalcoholic fatty liver
NASH: Non-alcoholic steatohepatitis
NCD: Normal control diet
PA: Palmitic acid
PMH: Mouse primary hepatocytes
ROS: Reactive oxygen species
siRNA: Small interfering RNA
TC: Total cholesterol
VLDL-TG: Very-low-density lipoprotein-associated triglyceride
WT: Wild type
3’UTR: 3′-untranslated regions
