# Supplementary material for: MicroRNA-379-5p regulates free cholesterol accumulation and relieves diet induced-liver damage in db/db mice via STAT1/HMGCS1 axis
Source: Mol Biomed. 2022 Aug 10;3:25. doi: 10.1186/s43556-022-00089-w (PMC9363541; doi:10.1186/s43556-022-00089-w)
Supplement: Supplementary file 1 — Additional file 1: Supplementary Fig. 1. MiR-379 negatively correlated with serum AST and ALT in clinic and mouse models. Supplementary Fig. 2. The TG content and H&E staining in db/db mice. Supplementary Table 1. Baseline characteristics of the study participants in GSE89632. Supplementary Table 2. MiR-379-5p regulates proteins involved in metabolism through KEGG analysis of the TMT-based quantitative proteomics. [file 43556_2022_89_MOESM1_ESM.docx]

**MicroRNA-379-5p regulates free cholesterol accumulation and relieves diet induced-liver damage in *db*/*db* mice via STAT1/HMGCS1 axis**

**Short Title**: MiR-379-5p modulates cholesterol synthesis

Yunxia Dong^1,2,#^, Chuwei Yu^1,2,#^, Ningning Ma^1,2,3^, Xiaoding Xu^1,2,4^, Qian Wu^1^, Henglei Lu^1^, Likun Gong^1,2^, Jing Chen*^,1,2^, Jin Ren*^,1,2,4^

^1^Center for Drug Safety Evaluation and Research, State Key Laboratory of Drug Research, Shanghai Institute of Materia Medica, Chinese Academy of Sciences, 501 Haike Road, Shanghai 201203, China

^2^University of Chinese Academy of Sciences, No.19A Yuquan Road Beijing 100049, China

^3^School of Life Science and Technology, ShanghaiTech University, 100 Haike Road, Shanghai 201210, China

^4^School of Chinese Materia Medica, Nanjing University of Chinese Medicine, Nanjing 210023, China

*Corresponding author: Jing Chen and Jin Ren, Center for Drug Safety Evaluation and Research, State Key Laboratory of Drug Research, Shanghai Institute of Materia Medica, Chinese Academy of Sciences, 501 Haike Road, Shanghai 201203, China.

E-mail: jingchen@simm.ac.cn (J. Chen), jren@cdser.simm.ac.cn (J. Ren)

Phone & fax number: +86-21-58382922

^#^ These two authors contributed equally to this work.


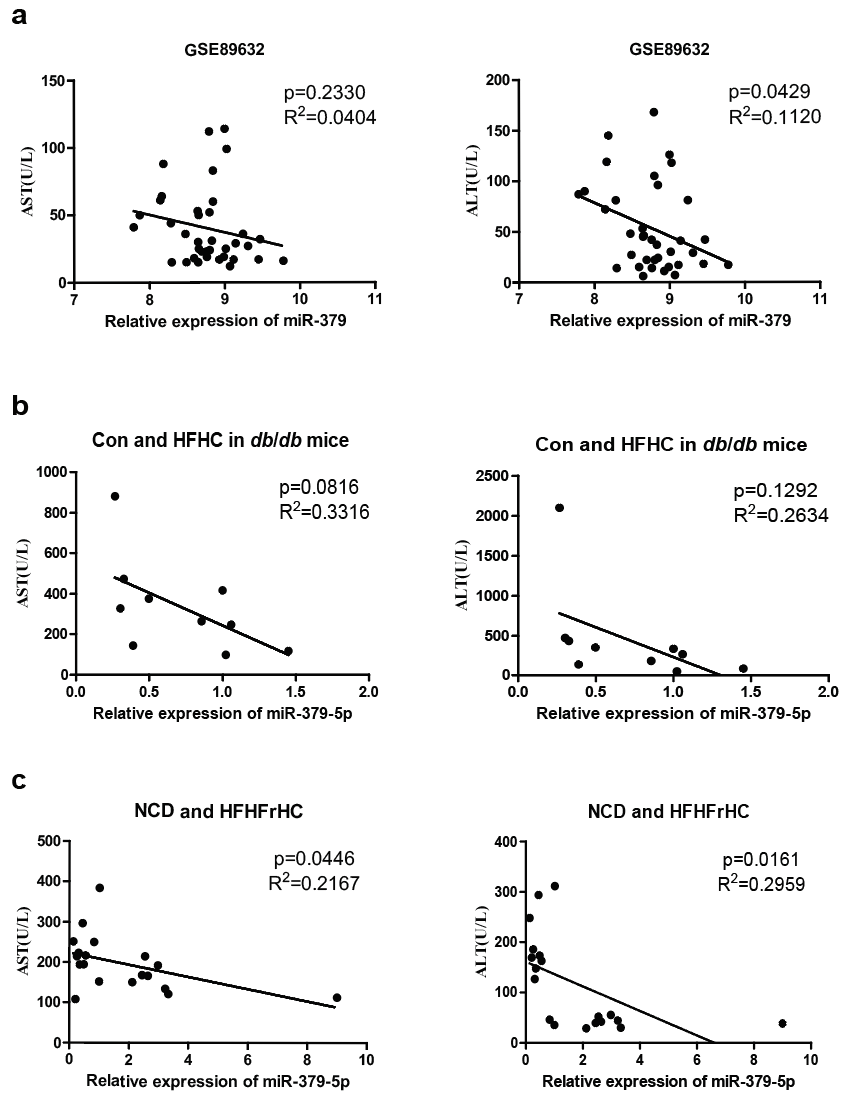


**Supplementary Fig. 1. MiR-379 negatively correlated with serum AST and ALT in clinic and mouse models. (a)** Relationship analysis of miR-379 and serum AST, ALT between healthy control (HC, *n* = 18) and NASH patients (*n* = 19). Data were derived from GEO database (GSE89632). **(b)** Relationship analysis of miR-379 and serum AST, ALT between *db*/*db* mice fed with a NCD or HFHC diet for 20 weeks. *n* = 5 in each group. **(c)** Relationship analysis of miR-379 and serum AST, ALT between C57BL/6J mice fed with a NCD or HFHFrHC diet for 20 weeks. *n* = 9-10 in each group.


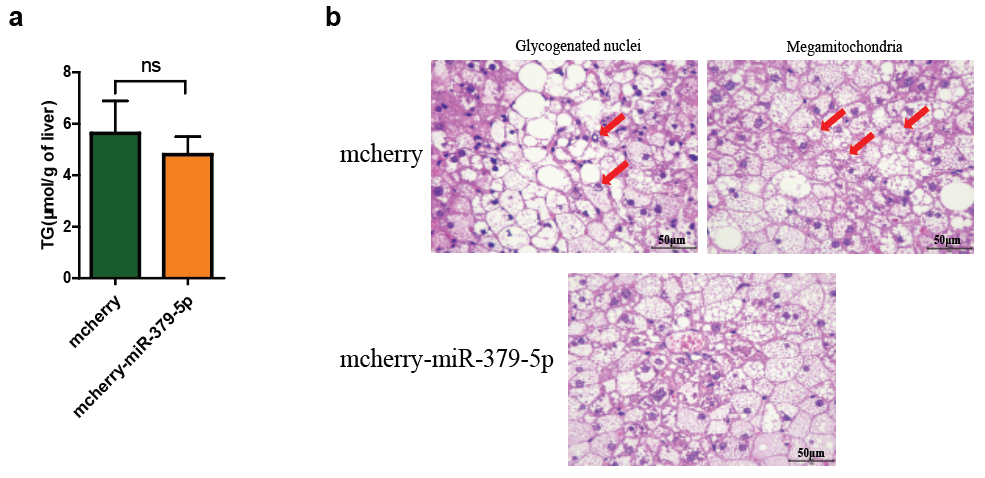


**Supplementary Fig. 2.** **The TG content and H&E staining in** ***db*/*db* mice. (a)** The TG content in the liver of mcherry and mcherry-miR-379-5p group with HFHC diet. **(b)** Representative H&E liver sections of mcherry and mcherry-miR-379-5p group with HFHC diet. Scale bar, 50 µm. Red arrow shows glycogenated nuclei (left) and megamitochondria (right).

**Supplementary Table 1: Baseline characteristics of the study participants in GSE89632.**

|  | HC | SS | NASH |
| --- | --- | --- | --- |
| **Clinical manifestation** |  |  |  |
| Sex (M/F) | 9/9 | 12/5 | 9/10 |
| Age (year) | 38.22±9.77 | 44.12±8.79 | 43.47±12.73 |
| Body mass index (kg/m^2^) | 26.29±4.39 | 28.89±4.48 | 31.77±5.45 |
| Waist (cm) | 90.99±11.11 | 100.14±10.49 | 104.19±10.37 |
| **Laboratory tests** |  |  |  |
| Aspartate transaminase (U/L) | 20.83±6.56 | 28.53±5.91 | 58.79±28.29 |
| Alanine transaminase (U/L) | 21.67±11.61 | 51.06±18.01 | 83.47±37.94 |
| Alkaline phosphatase (U/L) | 69.06±16.19 | 76.53±54.00 | 83.95±21.88 |
| Triglycerides (mmol/L) | 1.03±0.38 | 1.56±1.03 | 2.38±2.51 |
| Total cholesterol (mmol/L) | 4.91±0.92 | 4.83±1.12 | 4.98±1.10 |
| LDL cholesterol (mmol/L) | 3.10±0.70 | 3.03±0.91 | 2.88±0.91 |
| HDL cholesterol (mmol/L) | 1.35±0.37 | 1.08±0.24 | 1.16±0.29 |
| Fasting glucose (mmol/L) | 5.09±0.71 | 5.71±1.12 | 6.18±2.84 |
| Fasting insulin (pmol/L) | 79.07±152.81 | 71.67±47.68 | 198.14±219.48 |
| Homa-insulin resistance | 3.04±5.96 | 3.14±1.89 | 9.87±11.35 |
| Hemoglobin A1c | 0.06±0.01 | 0.05±0.00 | 0.06±0.01 |
| **NAFLD characteristics** |  |  |  |
| Steatosis (%) | 0.41±0.80 | 36.47±24.03 | 45.00±26.46 |
| Fibrosis (stage) | 0.00±0.00 | 0.00±0.00 | 1.95±1.51 |
| Lobular inflammation (severity) | 0.00±0.00 | 0.00±0.00 | 1.53±0.70 |
| Ballooning (intensity) | 0.00±0.00 | 0.00±0.00 | 1.32±0.48 |
| NAFLD activity score | 0.00±0.00 | 1.71±0.77 | 4.84±1.17 |

Data are presented as mean ± SEM. In HC group, 6 healthy living liver donors with fibrosis symptoms were excluded in Fig. 1a.

**Supplementary Table 2: MiR-379-5p regulates proteins involved in metabolism through KEGG analysis of the TMT-based quantitative proteomics**

| Protein names | Gene names | NC | miR-379-5p | Fold change | p-value |
| --- | --- | --- | --- | --- | --- |
| **28 up-regulated proteins involved in metabolic pathways** | | | | | |
| Purine nucleoside phosphorylase | PNP | 181823.59±  3015.60 | 256770.74±  8828.78 | 1.393236 | 0.000155 |
| Acyl-coenzyme A thioesterase 1; Acyl-coenzyme A thioesterase 2, mitochondrial | ACOT1 | 17030.50  ±  613.39 | 22070.43  ±  2790.42 | 1.249089 | 0.037828 |
| Glutamyl-tRNA (Gln) amidotransferase subunit C, mitochondrial | GATC | 19237.33±  1605.84 | 24616.56±  106.99 | 1.302444 | 0.004425 |
| Glycoprotein-N-acetylgalactosamine 3-beta-galactosyltransferase 1 | C1GALT1 | 825.99±  79.65 | 1085.48±  67.01 | 1.227865 | 0.012469 |
| UDP-glucose 6-dehydrogenase | UGDH | 240659.11±  12370.74 | 297592.53±  13118.82 | 1.299202 | 0.005439 |
| Deoxyuridine 5-triphosphate nucleotidohydrolase, mitochondrial | DUT | 113250.47±  7223.55 | 136470.52±  3576.94 | 1.215605 | 0.007547 |
| Glucosylceramidase | GBA | 6809.39±  398.88 | 9957.18±  727.17 | 1.469433 | 0.002771 |
| L-lactate dehydrogenase B chain; L-lactate dehydrogenase | LDHB | 1211933.68±  60547.81 | 1479210.54±  100675.46 | 1.257222 | 0.016952 |
| Argininosuccinate synthase | ASS1 | 30442.75±  2916.51 | 38565.05±  131.79 | 1.306068 | 0.008531 |
| Cytochrome P450 2S1 | CYP2S1 | 9535.76±  1449.61 | 12397.95±  254.81 | 1.255047 | 0.02809 |
| Multiple inositol polyphosphate phosphatase 1 | MINPP1 | 15382.49±  571.85 | 19127.94±  613.75 | 1.215544 | 0.001506 |
| Monofunctional C1-tetrahydrofolate synthase, mitochondrial | MTHFD1L | 17258.93±  503.65 | 24839.98±  993.72 | 1.433779 | 0.000297 |
| 3-hydroxybutyrate dehydrogenase type 2 | BDH2 | 14872.94±  369.11 | 20296.92±  804.21 | 1.337503 | 0.000446 |
| V-type proton ATPase 16 kDa proteolipid subunit | ATP6V0C | 157690.41±  16357.35 | 195423.59±  3767.10 | 1.294089 | 0.017637 |
| Myotubularin-related protein 14 | MTMR14 | 46736.65±  1844.20 | 55210.87±  3925.69 | 1.200863 | 0.027681 |
| Farnesyl pyrophosphate synthase | FDPS | 298328.79±  17968.36 | 386645.15±  24972.39 | 1.322292 | 0.00764 |
| Bifunctional methylenetetrahydrofolate dehydrogenase/cyclohydrolase, mitochondrial;NAD-dependent methylenetetrahydrofolate dehydrogenase;Methenyltetrahydrofolate cyclohydrolase | MTHFD2 | 27528.99±  1201.09 | 36520.46±  1456.52 | 1.302805 | 0.001178 |
| Sphingosine-1-phosphate lyase 1 | SGPL1 | 13982.17  ±  1572.41 | 18942.57±  2607.31 | 1.383766 | 0.047741 |
| Beta-1,4-glucuronyltransferase 1 | B4GAT1 | 62860.58±  2664.92 | 76790.99±  4200.34 | 1.250001 | 0.008337 |
| DNA polymerase delta subunit 2 | POLD2 | 1015.82  ±  180.46 | 1493.44±  90.76 | 1.460145 | 0.014908 |
| Aminopeptidase N | ANPEP | 18356.60±  716.59 | 23268.05±  552.10 | 1.261324 | 0.000713 |
| Lysosomal alpha-glucosidase;76 kDa lysosomal alpha-glucosidase;70 kDa lysosomal alpha-glucosidase | GAA | 15617.46±879.08 | 19266.90  ±  1791.46 | 1.301213 | 0.033936 |
| DNA-directed RNA polymerase II subunit RPB7 | POLR2G | 9558.57±  980.48 | 12594.63±  670.94 | 1.41857 | 0.011456 |
| Inositol-3-phosphate synthase 1 | ISYNA1 | 99519.15±  5986.72 | 126250.17±  2774.30 | 1.293395 | 0.002172 |
| Aconitate hydratase, mitochondrial | ACO2 | 208598.39±  6142.43 | 251310.11±  3471.13 | 1.205585 | 0.000468 |
| UDP-N-acetylhexosamine pyrophosphorylase;UDP-N-acetylgalactosamine pyrophosphorylase;UDP-N-acetylglucosamine pyrophosphorylase | UAP1 | 67890.07±  2363.22 | 86325.63±  4539.64 | 1.304449 | 0.003363 |
| 5-nucleotidase | NT5E | 9768.51±  856.31 | 15768.66±  619.54 | 1.700971 | 0.0006 |
| Long-chain-fatty-acid--CoA ligase 1 | ACSL1 | 41634.94±  3457.27 | 48607.77  ±  1186.10 | 1.200045 | 0.029814 |
| **3 down-regulated proteins involved in biosynthesis of unsaturated fatty acids** | | | | | |
| Acyl-CoA desaturase | SCD | 90117.19±  6753.81 | 59747.64±  4180.90 | 0.702785 | 0.002697 |
| Fatty acid desaturase 2 | FADS2 | 24696.49±  1117.03 | 17978.27±  1285.51 | 0.752115 | 0.0024 |
| Fatty acid desaturase 1 | FADS1 | 9807.87±  981.59 | 7063.26±  638.50 | 0.765305 | 0.015353 |

Data are presented as mean ± SD.
